# An Abundant and Renewable Potential Energy Source: Harvestable Energy under Vehicle Wheels

**DOI:** 10.1002/gch2.201800096

**Published:** 2019-02-20

**Authors:** Feng‐Chen Li

**Affiliations:** ^1^ Sino‐French Institute of Nuclear Engineering and Technology Sun Yat‐Sen University Zhuhai 519082 China

**Keywords:** energy harvesting, energy saving, renewable energy, vehicle wheels

## Abstract

Approaches for energy harvesting from rotating vehicle tires have been investigated for years; however, where the harvestable energy is actually generated has not been discussed so far. For the first time, the potentially harvestable energy under vehicle wheels, as a real energy source, is discovered. Estimations show that the global potentially harvestable energy from the vehicle wheels could be 3.26 × 10^11^ kW, equivalent to 14 500 times the installed capacity of the worldwide largest hydraulic power plant, Three Gorges Dam of China; 36.7% saving of a vehicle's fuel consumption could be achieved if this potential energy could be totally harvested. State‐of‐the‐art energy harvesting techniques can only extract a negligibly small amount of the energy under vehicle wheels, calling for revolutionary new energy harvesting techniques.

Energy sources are critically important to all the nations around the world and have long been among the hottest topics for scientific research. Development of energy sources is one of the common concerns for the mankind. Nuclear fusion fuel, deuterium, was thought of as the final solution of energy source for the mankind, since it can be extracted from the ocean and is nearly inexhaustible. However, it is still a long way to go to realize the commercial application of nuclear fusion energy. Coal, petroleum, natural gas, and nuclear fission fuel are the major energy sources that have been playing irreplaceable roles in developing the modern world. Hydraulic power, solar energy photovoltaic power, wind power, biomass energy, ocean tidal power, and gas hydrate are the main renewable energy sources that are playing or will play a subsidiary role to the major energy sources.

What else? Are there any other renewable energy sources undiscovered? The answer is undoubtedly “Yes.” In this paper, a not‐yet‐discovered (or neglected) renewable energy source, which is visible everywhere in the modern world, namely, energy under vehicle wheels, is reported. Although power harvesting from a rotating tire by a nanogenerator based on piezoelectric effect^1^ and triboelectric effect^2^, ^3^, ^4^, ^5^ or by an electromechanical wideband energy harvester^6^ has been realized for years, how much energy is indeed contained under the vehicle wheels (deformable tires) has never been discussed or discovered. As a renewable energy source, the power under the vehicle wheels has not yet been acknowledged.

Herein, for the first time, the energy contained under the vehicle wheels (due to the tires' deformation caused by the gross weight of vehicle together with passenger(s) and load) is discovered as a renewable energy source from the high‐density power viewpoint. It is derived that the conservative value of the estimated power accumulation under vehicles wheels all over the world can reach ≈10^11^ kW, which is more than 10 000 times the installed power of the Three Gorges Dam hydraulic power plant of China (the largest hydraulic power plant in the world). This idea about a real renewable energy source, from all aspects, is totally different from the explorations related to low‐density energy harvesting efforts so far.^1^, ^2^, ^3^, ^4^, ^5^, ^6^

Because the vehicle body is always moving in parallel to the ground surface without displacement in the direction of the gravitational acceleration (*g*), the power contained in the vehicle wheel system is not that apparent and thus has not been recognized as a renewable energy source so far. Now considering a wheel (note that, no matter it is a four‐wheel car or a ten‐wheel heavy truck, all the wheels are equivalently considered as one, and the vehicle weight is exerted on this one wheel) under a running vehicle at a speed *V*, the tire will be slightly flatted from the round shape at the interface between the tire and the ground due to the exerted weight of vehicle together with passenger(s) and load, as schematically shown in **Figure** **1**. The flatted part of the tire has a vertical displacement δ compared to the perfectly round state, which is caused by the counterforce, *F*_c_, from the ground equivalent to the value of the vehicle gravity force *Mg*, where *M* is the total mass of the vehicle together with passenger(s) and load. At one moment, the force *F*_c_ does work within displacement δ

(1) $$W_{i}=F_{c}\delta =Mg\delta$$

which is the actually existing and harvestable “unit energy” after the wheel rotates an arc length

(2) $$\Delta l=2R\;\cos^{-1}\left(1-\frac{\delta }{R}\right)$$

where *R* is the radius of the tire. The energy harvesting from vehicle wheels can most probably be realized with energy harvester arrays installed in the tires and each energy harvester unit (which can be a group of harvester elements arranged in the spanwise direction of the tire) is represented by a length similar to Δ*l*. The total power generated under the wheels of a vehicle running at a speed *V* can thus be obtained straightforwardly

(3) $$P=\frac{V}{\Delta l}Mg\delta =\frac{VMg\delta }{2R\;\cos^{-1}\left(1-\frac{\delta }{R}\right)}$$

**Figure 1:**
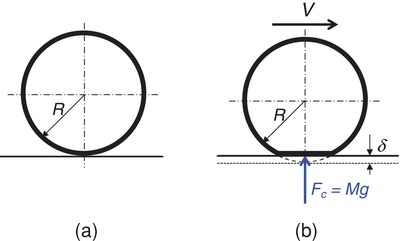
Schematic showing the state of tires under a running vehicle: a) perfectly round tire and b) slightly flat tire due to weight of vehicle together with passenger(s) and load.

Assigning values of 2000 kg and 0.32 m to *M* and *R*, respectively, should be meaningful for estimating the achievable power from vehicle wheels. Accordingly, the power *P*, as a function of *V* and δ, available from one vehicle can be obtained. As shown in **Figure** **2**, within the estimated reasonable ranges for *V* (40–120 km h^−1^) and δ (5–15 mm), the achievable power *P* is from 9.6 to 49.8 kW.

**Figure 2:**
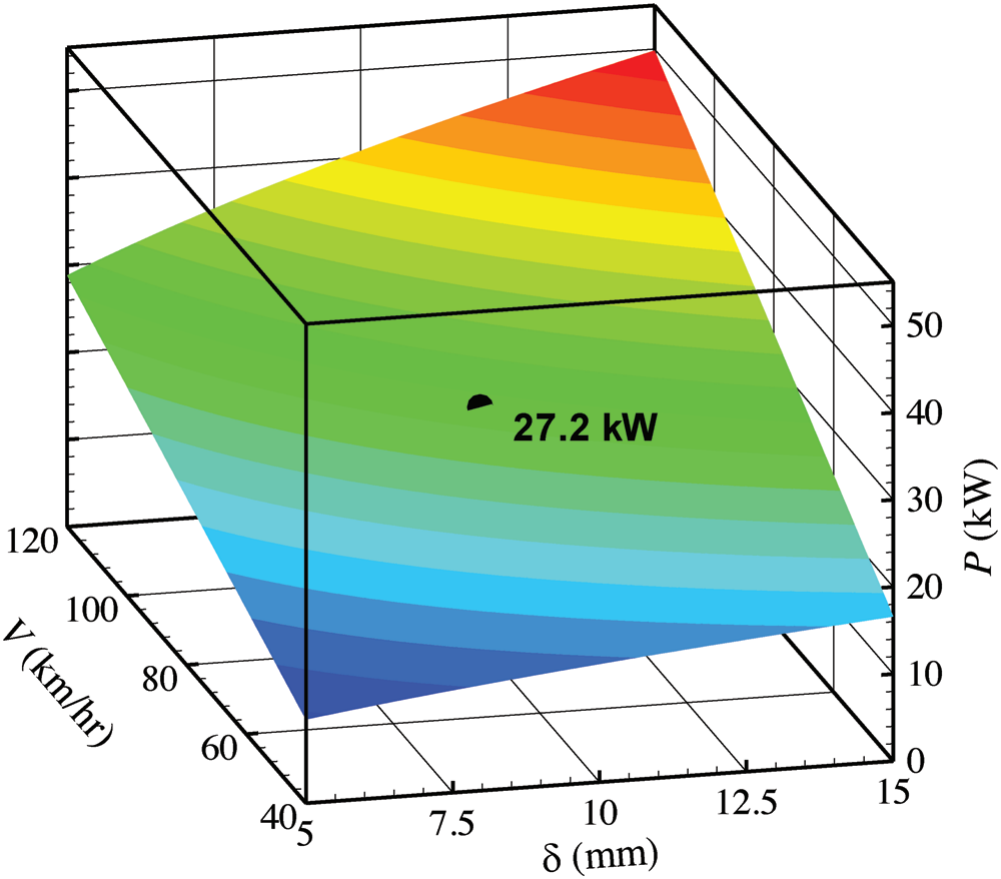
The estimated achievable power from vehicle wheels as a function of vehicle speed *V* and tire flattened depth δ. Estimations were made assuming the total mass of vehicle and passenger(s)/load *M* = 2000 kg and the radius of the tire *R* = 0.32 m. The single point at the center of the contour map exemplified an estimated harvestable power at *V* = 80 km h^−1^ and δ = 10 mm.

The energy saving effect can be roughly estimated with simple arithmetic as follows if the achievable power from the vehicle wheels could be thoroughly harvested. Figure 2 shows that *P* = 27.2 kW when the vehicle speed is 80 km h^−1^ and the tire flatted depth is 10 mm (at *M* = 2000 kg and *R* = 0.32 m). At these working conditions, assuming an average vehicle fuel (gasoline) consumption to be 10 L per 100 km, and taking the density of gasoline to be 0.725 kg L^−1^ and the calorific value of gasoline being 46 000 kJ kg^−1^, a vehicle will consume energy *E* = 3.335 × 10^5^ kJ after running 100 km. The total harvestable energy under the vehicle tires after running 100 km is arithmetically calculated to be *E*_t_ = 1.224 × 10^5^ kJ. It indicates that, if the potential energy from the vehicle wheels can be totally harvested, 36.7% energy saving of the vehicle can be achieved.

On the other hand, considering an estimated vehicle population of 1.2 billion all over the world^7^ and assuming those vehicles running on the road spontaneously (note that it is unrealistic for all the vehicles running at the same time, saying so herein is only to estimate the maximum potential capacity of harvestable power under the vehicle wheels) at the above assumed conditions, the total potentially available power under the vehicle wheels will be 3.26 × 10^11^ kW. Compared with the world's largest hydraulic power station, the Three Gorges Dam of China, with installed capacity of 2.25 × 10^7^ kW,^8^ the above estimated harvestable power under the vehicle wheels is equivalent to 14 500 times the installed capacity of the Three Gorges Dam hydraulic power plant, which is tremendously huge. Even considering a more realistic number of spontaneously running vehicles, for example, 1% of the vehicle population, the total harvestable power under the vehicle wheels can be equivalent to more than 100 times the installed capacity of the Three Gorges Dam, which is still tremendously huge. It indicates that the harvestable power under the vehicle wheels is a real and huge energy source. And as long as the vehicles keep running on the road, this energy source continuously exists and is renewable.

The reported energy harvesting techniques related to vehicle tires and in the way as mentioned earlier^1^, ^2^, ^3^ were all for the purpose of powering small wireless electric devices such as tire pressure monitoring sensors. Limited by the working principles, those explored energy harvesters are unable to extract high‐density power. The harvester power was only on the order of a few tens of milliwatts. Very recently, it was reported that at least 1.2 W of tire rotation energy could be obtained for a standard tire by means of compressible hexagonal‐structured triboelectric nanogenerators.^9^ Even so, it is still negligibly lower (<0.005%) than the potentially achievable power under vehicle wheels as discovered herein (Equation (3)). Apparently, there must be other approaches novelly invented in order to efficiently extract power from the energy source under vehicle wheels.

The theoretical derivation of the potentially available power under vehicle wheels presented in this report (Equation (3)) opens up huge renewable potential energy source. Note that there must be a significant discount to the estimated maximum potential capacity of harvestable power under the vehicle wheels. One major discount factor is the percentage characterizing how many vehicles run on the road spontaneously. The other major discount factor is the percentage characterizing the efficiency of energy harvesting techniques. The harvestable power could be a few orders lower than the maximum; nevertheless, it still deserves to be explored and extracted and calls for innovative small‐scale (milli‐ or microscale) energy harvesting techniques for high‐density power.

## Conflict of Interest

The author declares no conflict of interest.
